# Discovery Stories of RET Fusions in Lung Cancer: A Mini-Review

**DOI:** 10.3389/fphys.2019.00216

**Published:** 2019-03-19

**Authors:** Kengo Takeuchi

**Affiliations:** ^1^Division of Pathology, The Cancer Institute, Japanese Foundation for Cancer Research, Tokyo, Japan; ^2^Pathology Project for Molecular Targets, The Cancer Institute, Japanese Foundation for Cancer Research, Tokyo, Japan

**Keywords:** ALK, RET, fusion gene, FISH, lung cancer

## Abstract

In 2004, a chemical inhibitor of the kinase activity of EGFR was reported to be effective in a subset of lung cancer patients with activating somatic mutations of *EGFR*. It remained unclear, however, whether kinase fusion genes also play a major role in the pathogenesis of lung cancers. The discovery of the EML4-ALK fusion kinase in 2007 was a breakthrough for this situation, and kinase fusion genes now form a group of relevant targetable oncogenes in lung cancer. In this mini-review article, the discovery of REarrangement during Transfection fusions, the third kinase fusion gene in lung cancer, is briefly described.

## Introduction

Somatic mutations cause cancer via multiple mechanisms, including point mutations, insertions, deletions, and gene rearrangements. In non-small cell lung cancer (NSCLC), one of the most common causes of cancer-related deaths, these oncogenic mutations are usually mutually exclusive, and generally only a single major driver mutation is found in each case. In addition, such a cancer usually depends on the signal pathway stimulated by the principal oncogene for its survival (oncogene addiction) (Weinstein, 2002). In 2004, it was reported that a chemical inhibitor of the kinase activity of EGFR was effective in a subset of lung cancer patients with activating somatic mutations of *EGFR* (Lynch et al., 2004; Paez et al., 2004). NSCLC in which EGFR inhibitors are effective preferentially develop in Asian and non-smoker populations, generally lacking other targetable driver mutations (Paez et al., 2004; Pao et al., 2004; Shigematsu et al., 2005). In addition to *EGFR* mutations, kinase fusion genes have become a group of relevant oncogenes in NSCLC, because targeted inhibition of oncogenic kinase fusion proteins also leads to growth inhibition of the cancer cells and regression of the patient’s tumor.

Gene fusion was known to be a major mechanism of oncogenesis in hematopoietic neoplasms and sarcomas (Mitelman, 2000). Various types of fusion oncogenes were reported (Mitelman et al., 2007) after the identification of the BCR-ABL1 fusion kinase in chronic myelogenous leukemia (Bartram et al., 1983). In contrast, it remained unclear for a long time whether such fusion oncogenes also play a major role in the pathogenesis of epithelial tumors. The discovery of the EML4-ALK fusion kinase in NSCLC via inv(2)(p21p23) was a breakthrough in this scenario (Soda et al., 2007). Moreover, several small molecules, such as crizotinib (Kwak et al., 2010; Shaw et al., 2013) and alectinib (Seto et al., 2013; Takeuchi et al., 2016; Hida et al., 2017), showed improved survival outcomes in ALK fusion-positive NSCLC patients. These clinical successes suggested that targeting specific fusion kinases was a promising strategy also for treating carcinomas (epithelial cancers). Representative fusions in epithelial tumors are listed in Table 1.

**Table 1:** Representative fusion genes in epithelial tumors.

	Fusion gene	Hisological type
Lung carcinoma	*EML4-ALK*	Non-small cell carcinoma
	*TFG-ALK*	
	*KIF5B-ALK*	
	*KLC1-ALK*	
	*STRN-ALK*	
	*TPR-ALK*	
	*HIP1-ALK*	
	*SEC31A-ALK*	
	*BIRC6-ALK*	
	*KIF5B-RET*	
	*CCDC6-RET*	
	*NCOA4-RET*	
	*TRIM33-RET*	
	*RUFY2-RET*	
	*CUX1-RET*	
	*KIAA1468-RET*	
	*CD74-ROS1*	
	*SLC34A2-ROS1*	
	*SDC4-ROS1*	
	*EZR-ROS1*	
	*TPM3-ROS1*	
	*LRIG3-ROS1*	
	*GOPC (FIG)-ROS1*	
	*CCDC6-ROS1*	
	*MSN-ROS1*	
	*CD74-NTRK1*	
	*MPRIP-NTRK1*	
	*TPM3-NTRK1*	
	*TRIM24-NTRK2*	
	*BAG4-FGFR1*	
	*FGFR2-CIT*	
	*FGFR2-KIAA1967*	
	*FGFR3-TACC3*	
	*FGFR3-BAIAP2L1*	
	*SCAF11-PDGFRA*	
	*EZR-ERBB4*	
	*AXL-MBIP*	
	*TRIM4-BRAF*	
	*TRIM24-BRAF*	
	*SND1-BRAF*	
	*CD74-NRG1*	
	*VAMP2-NRG1*	
	*SLC3A2-NRG1*	
	*MAP4K3-PRKCE*	
	*BCAS3-MAP3K3*	
	*ERBB2IP-MAST4*	
	*KRAS-CDH13*	
	*APLP2-TNFSF11*	
	*ZFYVE9-CGA*	
	*TPD52L1-TRMT11*	
	*E2A-PBX1*	
	*KIF5B-MET*	
	*SPNS1-PRKCB*	
	*WASF2-FGR*	
	*ADCY9-PRKCB*	
Thyroid carcinoma	*CCDC6(H4)-RET*	Papillary carcinoma
	*TPM3-NTRK1*	
	*PRKAR1A-RET*	
	*NCOA4(ELE1)-RET*	
	*TFG-NTRK1*	
	*TPR-NTRK1*	
	*GOLGA5-RET*	
	*TRIM24-RET*	
	*TRIM33-RET*	
	*ERC1(RAB6IP2)-RET*	
	*KTN1-RET*	
	*RFG9-RET*	
	*PCM1-RET*	
	*RFP(TRIM27)-RET*	
	*AKAP9-BRAF*	
	*HOOK3-RET*	
	*EML4-ALK*	
	*PAX8-PPARG*	Follicular carcinoma
	*CREB3L2-PPARG*	
Breast carcinoma	*ETV6-NTRK3*	Secretary carcinoma
	*EML4-ALK*	
	*ARID1A-MAST2*	
	*GPBP1L1-MAST2*	
	*ZNF700-MAST1*	
	*NFIX-MAST1*	
	*TADA2A-MAST1*	
	*SEC16A-NOTCH1*	
	*SEC22B-NOTCH2*	
	*MAGI3-AKT3*	
Gastric carcinoma	*AGTRAP-BRAF*	
	*CD44-SLC1A2*	
	*CLDN18-ARHGAP26*	
	*SLC34A2-ROS1*	
Colorectal carcinoma	*TPM3-NTRK1*	
	*EML4-ALK*	
	*C2orf44-ALK*	
Prostate carcinoma	*TMPRSS2-ERG*	
	*SLC45A3-ERG*	
	*HERPUD1-ERG*	
	*NDRG1-ERG*	
	*SLC45A3-ELK4*	
	*TMPRSS2-ETV1*	
	*SLC45A3-ETV1*	
	*HERVK-ETV1*	
	*C15orf21-ETV1*	
	*HNRPA2B1-ETV1*	
	*ACSL3-ETV1*	
	*EST14-ETV1*	
	*HERVK17(FLJ35294)-ETV1*	
	*FOXP1-ETV1*	
	*TMPRSS2-ETV4*	
	*DDX5-ETV4*	
	*CANT1-ETV4*	
	*KLK2-ETV4*	
	*TMPRSS2-ETV5*	
	*SLC45A3-ETV5*	
	*ESRP1-RAF1*	
	*RAF1-ESRP1*	
	*SLC45A3-BRAF*	
Renal cell carcinoma	*PRCC-TFE3*	Xp11.2 translocation renal cell carcinoma
	*SFPQ-TFE3*	
	*NonO-TFE3*	
	*ASPSCR1-TFE3*	
	*CLTC-TFE3*	
	*t(3;X)(q23;p11.23)*	
	*Alpha(MALAT1)-TFEB*	
	*VCL-ALK*	
	*EML4-ALK*	
	*TPM3-ALK*	
	*STRN-ALK*	
Bladder carcinoma	*FGFR3-TACC3*	Urothelial carcinoma
	*FGFR3-BAIAP2L1*	
Salivary gland tumor	*CTNNB1-PLAG1*	Pleomorphic adenoma
	*LIFR-PLAG1*	
	*TCEA1-PLAG1*	
	*HMGA2-FHIT*	
	*HMGA2-NFIB*	
	*CHCHD7-PLAG1*	
	*HMGA2-WIF1*	
	*ETV6-NTRK3*	Secretory carcinoma
	*CRTC1-MAML2*	Mucoepidermoid carcinoma
	*CRTC3-MAML2*	
	*EWSR1-ATF1*	Clear cell carcinoma
	*EWSR1-CREM*	
	*MYB-NFIB*	Adenoid cytic carcinoma
	*MYBL1-NFIB*	

Receptor tyrosine kinases including ALK usually comprise an extracellular receptor domain, a transmembrane domain, and an intracytoplasmic tyrosine kinase domain. The receptor domain binds to ligands, resulting in dimerization of the kinase protein. Then, the dimerized proteins are autophosphorylated and stimulate the RAS-MAPK-ERK and PI3K-AKT pathways to promote cell proliferation, migration, and differentiation. A receptor tyrosine kinase gene rearrangement gives rise to the expression of the fusion kinase protein if the 5′-partner gene fuses with the 3′-kinase gene in an in-frame fashion. These fusion kinases can be oncogenic when they retain the kinase domain and are dimerized through the 5′ partner, because this dimerization mimics that of the wild-type receptor tyrosine kinases through ligand binding. Consequently, a fusion kinase is constitutively expressed, dimerized, and autoactivated, and its downstream signaling promotes cell proliferation and survival.

## Alk Fusion

ALK is a receptor tyrosine kinase that is not expressed in normal cells in adult mammals except for nerve cells. The most common mechanism of ALK overexpression and ALK kinase domain activation in neoplastic cells is the formation of a fusion protein with a partner through genomic rearrangement. In fact, ALK was first discovered in anaplastic large cell lymphoma (ALCL) in the form of a fusion protein, NPM1-ALK (Morris et al., 1994; Shiota et al., 1994). Other ALK fusion partners reported in ALCL are TFG, TPM3, TPM4, ATIC, RNF213, CLTC, MSN, MYH9, and TRAF (Hernandez et al., 1999; Lamant et al., 1999, 2003; Colleoni et al., 2000; Touriol et al., 2000; Meech et al., 2001; Tort et al., 2001; Cools et al., 2002; Feldman et al., 2013). NPM1-ALK is the most common ALK fusion in ALK-positive ALCL (70–80%), followed by TPM3-ALK (12–18%) (Tsuyama et al., 2017), and other fusions are rare. Except for ALCL, several hematopoietic neoplasms have been reported to have the following ALK fusion partners: CLTC, NPM1, SEC31A, SQSTM1, RANBP2, and EML4 in ALK-positive large B-cell lymphoma (Gascoyne et al., 2003; Van Roosbroeck et al., 2010; Takeuchi et al., 2011; Lee et al., 2014; Sakamoto et al., 2016); TPM3 in ALK-positive histiocytosis (Chan et al., 2008); and RANBP2 in myeloid leukemia (Maesako et al., 2014). In solid tumors, ALK fusions were identified in approximately 50% of inflammatory myofibroblastic tumor with the following fusion partners: TPM3, TPM4, CLTC, ATIC, CARS, SEC31A, RANBP2, PPFIBP1, FN1, TFG, EML4, LMNA, PRKAR1A, DCTN1, and RRBP1 (Lawrence et al., 2000; Bridge et al., 2001; Cools et al., 2002; Debelenko et al., 2003; Debiec-Rychter et al., 2003; Ma et al., 2003; Panagopoulos et al., 2006; Takeuchi et al., 2011; Lovly et al., 2014; Lee J.C. et al., 2017). Other ALK fusion-positive solid tumors include renal cancer (Debelenko et al., 2011; Marino-Enriquez et al., 2011; Sugawara et al., 2012; Kusano et al., 2016), colon cancer (Lin et al., 2009; Lipson et al., 2012; Stransky et al., 2014; Lee et al., 2015; Yakirevich et al., 2016), breast cancer (Lin et al., 2009), ovarian cancer (Ren et al., 2012), thyroid cancer (Cancer Genome Atlas Research Network, 2014; Kelly et al., 2014; McFadden et al., 2014; Perot et al., 2014; Stransky et al., 2014; Ji et al., 2015), and bladder cancer (Stransky et al., 2014). The frequencies are 1–2% in thyroid cancer (Cancer Genome Atlas Research Network, 2014; Kelly et al., 2014; McFadden et al., 2014; Ji et al., 2015) and less than 1% in kidney and colon cancers (Sugawara et al., 2012; Yakirevich et al., 2016). In NSCLC, EML4 is the most common partner of ALK. Although very rare, KIF5B, KLC1, TFG, STRN, PTPN3, HIP1, TPR, SEC31A, SQSTM1, DCTN1, and CRIM1 were also reported as an ALK fusion partner (Rikova et al., 2007; Takeuchi et al., 2009; Jung et al., 2012; Togashi et al., 2012; Majewski et al., 2013; Choi et al., 2014; Hong et al., 2014; Iyevleva et al., 2015; Kim et al., 2016; Tan et al., 2016).

## Ret Fusion

REarrangement during Transfection (RET) was identified by Takahashi et al. in 1985 as a proto-oncogene that underwent rearrangement during the transfection of DNA extracted from human T-cell lymphoma into NIH-3T3 cells (Takahashi et al., 1985). RET is a receptor tyrosine kinase encoded by a gene located on 10q11.22 (Ishizaka et al., 1989), and physiologically plays an important role in the development of neurons and kidneys. The first RET fusion in human cancer samples, CCDC6-RET, was identified in papillary thyroid carcinoma by Grieco et al. (1990). RET fusions are detected in 13–43% of papillary thyroid carcinomas (Kondo et al., 2006), and at least 12 RET fusions have been reported so far (Table 2).

**Table 2:** RET fusions in thyroid cancer.

RET fusion	Locus of the partner gene	Reference
CCDC6(H4)-RET	10q21.2	Grieco et al., 1990
PRKAR1A-RET	17q24.2	Bongarzone et al., 1993
NCOA4(ELE1)-RET	10q11.23	Bongarzone et al., 1994
GOLGA5-RET	14q32.12	Klugbauer and Rabes, 1999
TRIM24-RET	7q33-34	Klugbauer and Rabes, 1999
TRIM33-RET	1p13.2	
ERC1(RAB6IP2)-RET	12p13.33	Nakata et al., 1999
KTN1-RET	14q22.3	Salassidis et al., 2000
RFG9-RET	18q21-22	Klugbauer et al., 2000
PCM1-RET	8q21-22	Corvi et al., 2000
RFP(TRIM27)-RET	6p22.1	Saenko et al., 2003
HOOK3-RET	8p11.21	Ciampi et al., 2007

## Discovery of Ret Fusions in Lung Cancer

In 2012, the first RET fusion in lung cancer, KIF5B-RET, was reported independently by 4 groups from Korea (Ju et al., 2012), Japan (2 groups) (Kohno et al., 2012; Takeuchi et al., 2012), and the United States (Lipson et al., 2012). Ju et al. (2012) examined tissue and peripheral blood samples from a 33-year-old Korean never-smoking male with lung adenocarcinoma. The patient was negative for *EGFR* and *KRAS* mutations, and the *EML4-ALK* fusion gene, which were the three well-known driver mutations in lung adenocarcinoma at that time. Fifty-two fusion transcripts were called by transcriptome analysis in the patient’s adenocarcinoma. Out of 52 fusions, they could detect a corresponding genomic rearrangement only for *KIF5B-RET* fusion (KIF5B exon 16;RET exon 12 fusion variant. K16;R12) by whole genome sequencing. Additionally, they performed transcriptome analysis in 5 lung adenocarcinomas that were negative for *EGFR* and *KRAS* mutations and *EML4-ALK*, and identified one more case with *KIF5B-RET* fusion transcript (K15;R12). Furthermore, they found another KIF5B-RET-positive case (K23;R12) in 15 “double-negative (negative for *EGFR* mutation and *EML4-ALK* but *KRAS* status unknown)” lung adenocarcinomas by RT-PCR. Based on their detection rate, they estimated that the fusion might exist in approximately 6% of lung adenocarcinomas.

The following three studies were published in the same issue of the same journal, reflecting the “fusion kinase discovery race in major carcinomas” in those days. In the three studies, the frequency and oncogenicity of KIF5B-RET were more specifically evidenced, and growth inhibition analyses using cell lines and RET inhibitors were performed. Kohno et al. (2012) at the National Cancer Center researchers in Japan performed whole-transcriptome sequencing of 30 lung adenocarcinomas to identify new fusion genes that could be targeted for therapy. As a result, they discovered a *KIF5B-RET* fusion transcript in 1 out of 30 cases. In addition, 289 Japanese lung adenocarcinomas were screened by RT-PCR and Sanger sequence analyses, and the *KIF5B-RET* fusion gene was identified in 5 cases. In total, they identified 6 KIF5B-RET-positive cases out of 319 lung adenocarcinomas (1.9%), and 4 fusion variants in these 6 tumors. They also examined lung adenocarcinomas in the United States and Norway, and detected a *KIF5B-RET* transcript in one of the 80 (1.3%) subjects from the United States, but not in the 34 from Norway. They exogenously expressed a *KIF5B-RET* transcript (*KIF5B* exon 15;*RET* exon 12 variant. K15;R12) in the H1299 human lung cancer cell line and showed that Tyr905 was phosphorylated in the absence of serum stimulation. This phosphorylation was suppressed by vandetanib, a tyrosine kinase inhibitor to several receptor tyrosine kinases, including RET. They also showed that expression of exogenous KIF5B-RET induced morphological transformation and anchorage-independent growth of NIH-3T3 cells, which was suppressed by vandetanib.

Lipson et al. (2012) analyzed genomic DNA extracted from 24 formalin-fixed paraffin-embedded (FFPE) specimens of NSCLC by capture sequencing targeting 2,574 coding exons of 145 cancer-relevant genes and 37 introns of 14 frequently rearranged genes in cancer. They identified a *KIF5B-RET* transcript (K15;R12), generated via an 11,294,741-bp pericentric inversion on chromosome 10 in a lung adenocarcinoma from a 44-year-old never-smoking man of European ancestry. They detected *KIF5B-RET* fusions by RT-PCR in 1 of 121 (0.8%) European-ancestry and 9 of 405 (2%) Asian subjects, all of whom were never or limited former smokers. They estimated an overall occurrence rate of 2.0% (95% CI 0.8–3.1%). Four transcript variants were reported by them: K15;R12, K16;R12, K22;R12, and K15;R11. Ba/F3 cells, which are dependent on interleukin-3 (IL-3) for growth, that expressed KIF5B-RET were transformed and lived without IL-3. The cells were sensitive to sunitinib, sorafenib, and vandetanib, which are multi-target kinase inhibitors that inhibit RET.

Unlike the above-mentioned three studies, Takeuchi et al. (2012) identified KIF5B-RET fusions without next-generation sequencing analyses, but with traditional methods. They established an integrated platform of conventional histopathology and molecular pathology to identify fusion genes in various types of cancer. They performed fluorescence *in situ* hybridization (FISH) with their laboratory-made probes on tissue microarrays of various types of cancers. Using lung cancer tissue microarrays containing 1,528 samples, rearrangement of *KIF5B* was examined by a split FISH assay to discover new fusions, because they previously identified KIF5B-ALK fusions in lung cancer (Takeuchi et al., 2009) and thus hypothesized that KIF5B might fuse to other kinases in lung cancer. Twenty-four *KIF5B* split FISH-positive tumors were identified; among them, a *KIF5B-RET* transcript (K23;R12) was identified by 3′ rapid amplification of cDNA ends (RACE). Then, 22 *RET* rearrangement-positive tumors were identified in 1,528 lung cancers by RET split FISH. Among the 22 cases, 12 KIF5B-RET-positive tumors were identified through a multiplex RT-PCR system that captures all possible KIF5B-RET fusions: 8 cases with K15;R12, and one case each with the K16;R12, K22;R12, K23;R12, and K24;R11. The presence of inv(10)(p11.22q11.2) was supported by a *KIF5B-RET* fusion FISH assay in all 12 of these tumors. In lung cancer, they also identified CCDC6-RET, which is the first RET fusion identified in thyroid cancer (Grieco et al., 1990). In a routine pathology diagnosis during the study period, a pathologist in the group encountered an adenocarcinoma with a mucinous cribriform pattern that is a histopathological marker for the presence of EML4-ALK (Inamura et al., 2008). The case was, however, negative for ALK fusion and was positive for CCDC6-RET, as determined by FISH and inverse RT-PCR. In the remaining 10 tumors, another CCDC6-RET-positive tumor was identified by RT-PCR. In total, 14 RET fusion-positive tumors (13 out of the 1,528 tumors tested, and one additional tumor found through a routine pathology diagnostic service) were identified. RET fusions existed in 0.9% (13 out of 1,482) of the NSCLCs and 1.2% (13 out of 1,119) of the adenocarcinomas. The researchers demonstrated the oncogenicity of all the 5 KIF5B-RET fusion variants they identified through a focus formation assay and a mouse subcutaneous transplantation assay using NIH-3T3 cells expressing each KIF5B-RET variant. KIF5B-RET (K15;R12) transfected Ba/F3 cells grew in the absence of IL-3. Vandetanib inhibited the proliferation of cells expressing K15;R12 but not the proliferation of cells expressing EML4-ALK.

To date, at least 15 RET fusions have been reported in NSCLC including KIF5B-RET (Ju et al., 2012; Kohno et al., 2012; Lipson et al., 2012; Takeuchi et al., 2012), CCDC6-RET (Takeuchi et al., 2012), NCOA4-RET (Wang et al., 2012), TRIM33-RET (Drilon et al., 2013), RUFY2-RET (Zheng et al., 2014), CUX1-RET (Lira et al., 2014), KIAA1468-RET (Nakaoku et al., 2014), CLIP1-RET (Drilon et al., 2016), ERC1-RET (Drilon et al., 2016), MYO5C-RET (Lee S.H. et al., 2017), EPHA5-RET (Gautschi et al., 2017), PICALM-RET (Gautschi et al., 2017), FRMD4A-RET (Velcheti et al., 2017), KIF13A-RET (Zhang et al., 2018), and WAC-RET (Velcheti et al., 2018; Table 3). Most cases of RET fusion-positive NSCLCs are adenocarcinoma, although some authors reported non-adenocarcinoma cases including adenosquamous cell carcinoma (Wang et al., 2012; Song et al., 2017) and squamous cell carcinoma (Cai et al., 2013). In RET fusion-positive adenocarcinomas, specific histological features were not identified, although several characteristic features like cytoplasmic mucin production were detected (Tsuta et al., 2014). Driver mutations in other genes including *EGFR, KRAS, HER2, BRAF, ALK*, and *ROS1* are rare.

**Table 3:** RET fusions in lung cancer.

RET fusion	Locus of the partner gene	End exon of the partner gene	Start exon of RET	Reference	#cases	age	Sex	Country/race	Histopathology	EGFR mutation	KRAS mutation	Other driver mutation
KIF5B-RET	10p11.22	–	–	Ju et al., 2012	3	–	–	Korea	Adenocarcinoma	0/3	0/2	Negative for *EML4-ALK*
		–	–	Kohno et al., 2012	7	–	–	6 Japan, 1 United States	Adenocarcinoma	0/7	0/7	Negative for *HER2* mutation and *ALK* rearrangement
		–	–	Takeuchi et al., 2012	12	–	–	Japan	Adenocarcinoma	0/12	0/12	Negative for *ALK* and *ROS1* rearrangements
		–	–	Lipson et al., 2012	12	–	–	NA	Adenocarcinoma	0/12	0/12	Negative for *ERBB2* and *BRAF* mutations, *EML4-ALK*, and *ROS1* rearrangements
CCDC6-RET	10q21.2	1	12	Takeuchi et al., 2012	2	–	–	Japan	Adenocarcinoma	0/2	0/2	Negative for *ALK* and *ROS1* rearrangements
NCOA4-RET	10q11.23	6	12	Wang et al., 2012	1	80	F	NA	Adenocarcinoma	0/1	0/1	Negative for *ALK* rearrangement
TRIM33-RET	1p13.2	14	12	Drilon et al., 2013	1	41	F	Caucasian	Adenocarcinoma	0/1	0/1	Negative for *NRAS, BRAF, HER2, PIK3CA, MAP2K1*, and *AKT* mutations and *ALK* and *ROS1* rearrangements
RUFY2-RET	10q21.3	9	12	Zheng et al., 2014	1	NA	NA	NA	Adenocarcinoma	0/1	0/1	Negative for aberrations in otder driver genes detectable witd tde system
CUX1-RET	7q22.1	10	12	Lira et al., 2014	1	49	M	Korea	Adenocarcinoma (solid)	0/1	0/1	Negative for *ALK* and *ROS1* rearrangements
KIAA1468-RET	18q21.33	10	12	Nakaoku et al., 2014	1	62	M	Japan	Adenocarcinoma (invasive musinous)	0/1	0/1	Negative by RNA sequencing
CLIP1-RET	12q24.31	NA	NA	Drilon et al., 2016	1	NA	NA	NA	NA	NA	NA	NA
ERC1-RET	12p13.33	NA	NA	Drilon et al., 2016	1	NA	NA	NA	NA	NA	NA	NA
MYO5C-RET	15q21.2	25	12	Lee S.H. et al., 2017	1	NA	NA	NA	Adenocarcinoma	0/1	NA	Negative for *ALK* rearrangement
EPHA5-RET	4q13.1-q13.2	NA	NA	Gautschi et al., 2017	1	NA	NA	NA	NA	NA	NA	NA
PICALM-RET	11q14.2	NA	NA	Gautschi et al., 2017	1	NA	NA	NA	NA	NA	NA	NA
FRMD4A-RET	10p13	12	12	Velcheti et al., 2017	1	65	F	white	Non-small cell carcinoma (positive for TTF1 and napsin A, negative for p63 and CK5/6)	0/1	0/1	Negative for *ALK* and *ROS1* rearrangements
KIF13A-RET	6p22.3	18	12	Zhang et al., 2018	1	74	F	China	Adenocarcinoma	0/1	NA	Negative for *ALK* and *ROS1* rearrangements
WAC-RET	10p12.1	3	12	Velcheti et al., 2018	1	62	F	White	Adenocarcinoma	0/1	0/1	Negative for *ALK* rearrangement

## Concluding Remarks

REarrangement during Transfection-positive lung cancers constitute a small subset of lung adenocarcinomas showing clinicopathological features similar to those of other fusion kinase-positive lung cancers. Since their discovery, several trials for RET-positive lung cancer have been conducted using kinase inhibitors including vandetanib, cabozantinib, sorafenib, sunitinib, lenvatinib, ponatinib, and dovitinib. Although some clinical benefits were observed, efficacy was limited compared with that shown by EGFR and ALK inhibitors. The above-mentioned agents used in earlier trials are multi-kinase inhibitors, and are notably more effective to VEGFR, EGFR, and KIT than RET. Therefore, off-target dose limiting toxicity caused frequent dose reduction and discontinuation. RET inhibitors with more specificity and hence less off-target toxicity are currently undergoing clinical and preclinical development.

## Author Contributions

The author confirms being the sole contributor of this work and has approved it for publication.

## Conflict of Interest Statement

The author declares that the research was conducted in the absence of any commercial or financial relationships that could be construed as a potential conflict of interest.
